# Ethics of the Use of Social Media as Training Data for AI Models Used for Digital Phenotyping

**DOI:** 10.2196/59794

**Published:** 2024-07-17

**Authors:** Aditi Jaiswal, Aekta Shah, Christopher Harjadi, Erik Windgassen, Peter Washington

**Affiliations:** 1 Department of Information and Computer Sciences University of Hawaii at Manoa Honolulu, HI United States; 2 Salesforce San Francisco, CA United States; 3 Department of Computer Science University of California, Berkeley Berkeley, CA United States; 4 Department of Computer Science and Engineering University of California, Riverside Riverside, CA United States

**Keywords:** social media analytics, machine learning, ethics, research ethics, consent, scientific integrity

## Abstract

Digital phenotyping, or personal sensing, is a field of research that seeks to quantify traits and characteristics of people using digital technologies, usually for health care purposes. In this commentary, we discuss emerging ethical issues regarding the use of social media as training data for artificial intelligence (AI) models used for digital phenotyping. In particular, we describe the ethical need for explicit consent from social media users, particularly in cases where sensitive information such as labels related to neurodiversity are scraped. We also advocate for the use of community-based participatory design principles when developing health care AI models using social media data.

Community-based participatory research and human-centered design are central to research that aims to advance health equity [1]. While participatory design is a well-known framework that is increasingly, although not yet widely, used for research in areas such as interventions development [2] and partnered science, there is a dearth of research that builds artificial intelligence (AI) models for health in a manner that is grounded in community-based principles. The lack of community guidance early in the AI development process may lead, inadvertently, to models that are unethical despite being formally approved by an institutional review board (IRB). In particular, we discuss the topic of consent, which we argue spans at least two parts of the AI development process: (1) consent to build the AI model, which can be determined through participatory design sessions with the community that the AI model is meant to serve; and (2) consent to use an individual’s data within the training process of the model, which can be obtained through explicit consent procedures.

We discuss these gaps in community-based research for AI, with a particular focus on the development of social media–based screening tools for underserved communities, especially neurodiverse populations. Using social media for the quantification of characteristics or traits of an individual is a form of digital phenotyping, a method that can work with a broad range of data sources [3]. While the increasing availability of public data trails on social media can lead to predictive models that are possibly useful for creating positive good for health outcomes, the unrestricted use of these data poses the risk of training machine learning models on user-generated content without the explicit consent of the people who generated the data. Furthermore, the release of such models has the potential to lead to unintended consequences and possibly harm.

Social media platforms have emerged as a popular data source for several research domains, including for screening and surveillance broadly in psychiatry and behavioral sciences [4-6], sometimes with the help of AI. Government agencies such as the National Institutes of Health (NIH) in the United States encourage research that uses existing data streams, including social media, to provide actionable insights for conditions such as substance use [4-7]. However, several thought leaders are noting that such research must be carefully performed so as to not scrape data from the internet without the consent of the end users [8]. Some recent papers in social media analytics have been careful to obtain explicit consent from users participating in the study or to only conduct the analysis on anonymized data feeds. The NIH has started to prioritize funding research that addresses these ethical challenges [4-8]. In late 2023, The White House highlighted the need for ethical AI practices via its list of “Voluntary AI Commitments” created for companies [9] that are also highly relevant to noncommercial research, including guidelines such as prioritizing “research on societal risks posed by AI systems” and protecting privacy.

This conversation intersects strongly with the discourse around the training procedures of large language models, many of which have been trained on web data without user consent. Over the last few years, generative AI has revolutionized the field of AI by demonstrating remarkable capabilities from generating human-like text to creating art and music. These models require massive amounts of pretraining data collected from various public forums. However, there have been numerous examples of popular language models being trained with web data without explicit user consent or consent that was hidden away in terms and conditions. For example, users were concerned that Google was famously suspected of training Bard/Gemini using Gmail data without consent from end users, although Google denies these claims. Similarly, OpenAI has trained ChatGPT using data from users’ conversation histories. These cases raise questions about how our social contracts may have changed and what users inadvertently opt for when signing up on social media. Although OpenAI has provided the option to opt out of data retention, the default opt-in option raises privacy and data concerns.

The issue of data consent is particularly salient for vulnerable and marginalized groups. There are several instances of well-known misuse of data for scientific purposes. HeLa cells, named after Henrietta Lacks, are well known in the field of biology and have contributed greatly to progress in science. However, HeLa cells were commercialized, leading to financial gains without compensation or even an acknowledgement of Henrietta Lacks’ contributions. Another notable example is the historical misuse of Indigenous DNA through repeated lack of informed consent by members of Indigenous populations. 

In light of these reflections and the evolving discussions around AI ethics, we have elected to make some significant amendments to our recently published Twitter analysis paper on the use of the #ActuallyAutistic hashtag on Twitter for training a machine learning model that could serve as a screening tool for autism [10]. This paper serves as an example of what is possible with AI and social media in today’s tech ecosystem, and we provide a word of caution for creators of such models to think through how such models may be misused and interpreted by the community that they were built to serve. Models meant to help the autistic community should be built in collaboration with the community from the onset of the ideation and development process or should be led by autistic individuals. We hope that our decision to delete our data set and model can serve as a template for other researchers.

We would like to highlight two important closing thoughts. First, approval by an IRB does not necessarily translate to an ethical study. Some institutions are creating ethical review boards to provide an additional layer of ethical review of studies. Second, while many areas of health-related research are guided by community-based participatory principles, such practices are not as commonplace in research at the intersection of health, social media, and AI. Speaking with impacted communities helps verify assumptions and provides input into methods design and analysis, leading to more robust conclusions for future research.

## Abbreviations

AI: artificial intelligence
IRB: institutional review board
NIH: National Institutes of Health
